# SRPK1/AKT axis promotes oxaliplatin-induced anti-apoptosis via NF-κB activation in colon cancer

**DOI:** 10.1186/s12967-021-02954-8

**Published:** 2021-06-30

**Authors:** Jing-Qiang Huang, He-Feng Li, Jing Zhu, Jun-Wei Song, Xian-Bin Zhang, Peng Gong, Qiu-Yu Liu, Chun-Hui Zhou, Liang Wang, Li-Yun Gong

**Affiliations:** 1 Guangdong Key Laboratory for Genome Stability and Human Disease Prevention, Department of Biochemistry and Molecular Biology, Health Science Center, Shenzhen University, Shenzhen, Guangdong 518060 P. R. China; 2 School of Biomedical Engineering, Health Science Center, Shenzhen University, Shenzhen, Guangdong 518060 P. R. China; 3 Guangdong Provincial Key Laboratory of Regional Immunity and Diseases, Department of Pathogen Biology, Health Science Center, Shenzhen University, Shenzhen, 518060 Guangdong China; 4 Department of General Surgery, Shenzhen University General Hospital, Shenzhen, Guangdong 518060 P. R. China; 5 Department of Pathology, Henan Provincial People‘s Hospital, Zhengzhou, Henan 450003 P. R. China; 6 Department of Pathology, Guangzhou Health Science College, Guangzhou, Guangdong 510520 P. R. China; 7 Department of Cell Biology and Medical Genetics, Health Science Center, Shenzhen University, Shenzhen, Guangdong 518060 P. R. China

**Keywords:** SRPK1, NF-κB activation, Colon cancer, Anti-apoptosis, Oxaliplatin resistance

## Abstract

**Background:**

Colorectal cancer is the third most common diagnosis. Oxaliplatin is used as first-line treatment of colon cancer. However, oxaliplatin resistance greatly reduces its therapeutic effect. SRPK1 involves in pre-mRNA splicing and tumorigenesis. How SRPK1 mediates drug resistance in colon cancer is unknown.

**Methods:**

The expression of SRPK1 was analyzed in the TCGA and the CPTAC pan-cancer samples and detected in colon cancer cell lines and tissues by IHC and western blot. The MTT and TUNEL assay were used to verify the anti-apoptosis ability of colon cancer cell. The activation of NF-κB was determined by luciferase assay and qRT-PCR. AKT, IKK, IκB and their phosphorylation level were verified by western blot.

**Results:**

We found that *SRPK1* expression was the second highest in TCGA and the CPTAC pan-cancer samples. The mRNA and protein levels of SRPK1 were increased in tissues from patients with colon cancer. SRPK1 was associated with clinical stage and TNM classifications in 148 cases of colon cancer patients. High SRPK1 levels correlated with poor prognosis (*p* < 0.001). *SRPK1* overexpression enhanced the anti-apoptosis ability of colon cancer cells, whereas *SRPK1* silencing had the opposite effect under oxaliplatin treatment. Mechanistically, SRPK1 enhances IKK kinase and IκB phosphorylation to promote NF-κB nuclear translocation to confer oxaliplatin resistance.

**Conclusions:**

Our findings suggest that SRPK1 participates in colon cancer progression and enhances the anti-apoptosis capacity to induce drug resistance in colon cancer cells via NF-κB pathway activation, and thus might be a potential pharmaceutically target for colon cancer treatment.

**Supplementary Information:**

The online version contains supplementary material available at 10.1186/s12967-021-02954-8.

## Background

Colon cancer is the third most common cancer type in the world [1]. More than 1 million people are diagnosed with colon cancer every year [2]. Until now, chemotherapy has been the first option for most of patients with colon cancer. Oxaliplatin and doxorubicin represent a major class of chemotherapeutic drugs that can induce DNA damage in cancer cells [3]; however, chemoresistance is a major obstacle for anti-cancer treatment. Therefore, identifying the resistance-associated protein kinase that participates in oxaliplatin treatment resistance in colon cancer is urgent and important.

Serine-arginine protein kinase 1 (SRPK1) is a protein kinase that can phosphorylate serine-arginine (SR) rich splicing factors (SRSFs) reversibly [4]. Once SR domains are phosphorylated by SRPK1, SRSFs can participate in biological processes, including pre-mRNA splicing [5], translation regulation [6, 7], and genomic stability [8, 9]. In addition, SRPK1 is involved in the progression of many types of cancer, including breast cancer, prostate cancer, lung cancer, and melanoma, and the expression of its downstream targets are reported to be increased significantly [10–14]. Gong et al. reported that SRPK1 activates the Wnt/β-catenin pathway to promote a stem cell-like phenotype in non-small cell lung cancer (NSCLC) [14]. Wang et al. reported that SRPK1 interferes with PH domain and leucine rich repeat protein phosphatase 1 (PHLPP1)-mediated dephosphorylation of protein kinase B (AKT). Low expression of SRPK1 is unable to recruit PHLPP1, which is the phosphatase of AKT, whereas PHLPP1 might be titrated away from AKT by high expression of SRPK1 [13]. Controversially, SRPK1 is related to both chemotherapy sensitivity and resistance in many cancers, including lung, prostate, male germ cell, retinoblastoma, pancreas, colon, and breast cancer [15–19]. SRPK1 autophosphorylation enhances its kinase activity and nuclear translocation [20]. The modification of SRPK1 is involved in multiple drug-resistance signaling cascades, including osmotic stress and the epidermal growth factor (EGF)-EGF receptor (EGFR)-AKT pathway [20–22]. Taken together, SRPK1 plays an important role in tumor-associated pathway activation, which might enhance drug-resistance.

Anti-apoptosis plays a key role in cancer cell escape from treatment benefits. The nuclear factor kappa B (NFκB) transcription factor can regulate immune system components expression [23] and has been reported to regulate proteins that inhibit apoptosis and promote proliferation, which is associated with cancer [24]. The NF-κB family includes five members: RelA (p65), RelB, c-Rel, p105/p50, and p100/p52. The transcription activation domains of NF-κB are only found in p65, RelB, and c-Rel [25]. When the NF-κB pathway is inactivated, the inhibitor of κB protein (IκB) combines with p65/p50 heterodimers, thus keeping NF-κB in the cytosol without nucleocytoplasmic shuttling. When the cell receives a signal from the extracellular environment, IκBα will be phosphorylated by the inhibitor of κB kinase (IKK), followed by ubiquitination and degradation in proteasomes [26–28]. Cells resistant to doxorubicin have been reported to induce the NF-κB signaling pathway, resulting in transactivation of potent anti-apoptosis genes [3]. Aberrant NF-κB activation has been observed in multiple cancer types [29], which provides a potential strategy for reversing chemoresistance by targeting the NF-κB pathway.

In the present study, we found that SRPK1 is overexpressed in tissues from patients with colon cancer and in colon cancer cells. SRPK1 overexpression was significantly associated with clinical stage and the tumor-node-metastasis (TNM) classification of paraffin embedded sections from patients with colon cancer’. Upregulation of SRPK1 expression increased the anti-apoptosis ability, whereas downregulation of SRPK1 expression increased the pro-apoptotic sensitivity of colon cancer cells. Moreover, we found that SRPK1 could enhance IKK and IκB phosphorylation to promote NF-κB translocation from the cytoplasm to the nucleus and activate the NF-κB pathway. Furthermore, AKT phosphorylation increased in SRPK1 overexpression cell lines. Conversely, silencing SRPK1 decreased AKT phosphorylation. In addition, the phosphorylation of IKK and IκB were inhibited by an AKT inhibitor in SRPK1 overexpression colon cancer cell lines. Taken together, these findings reveal that aberrant SRPK1 expression activates the NF-κB pathway via AKT, which participates in the anti-apoptosis process of colon cancer. Thus, our findings suggest that SRPK1 is significantly associated with colon cancer and might be a potential pharmaceutical target to treat colon cancer.

## Methods

### Cell culture

Colon cancer cell lines (SW620, LOVO, HCT-8, HT-29, SW480 and HCT-116) and 293 T cell were purchased from the American Type Culture Collection (ATCC, Manassas, VA, USA) and the human normal intestinal epithelial cell line NCM-460 was obtained from Dr. Ying Ying (Shenzhen University, Shenzhen, China). All cells were cultured in Dulbecco’s Modified Eagle’s Medium (DMEM, Invitrogen, Carlsbad, CA, USA) with 10% fetal bovine serum (FBS, Gibco, Waltham, MA, USA) and 1% penicillin/streptomycin (15,140,122, Gibco) at 37 °C in a 5% CO_2_ atmosphere. We used 0.25% trypsin (25,200,056, Gibco) to passage the cell lines when they grew to 90% confluence. All cell lines used in this study were authenticated using the short tandem repeat (STR) method (Additional file 1: Table S4) and verified as being free of mycoplasma contamination using polymerase chain reaction (PCR).

### RNA extraction, reverse transcription, and qRT-PCR analysis

Total RNA from colon cancer cells was extracted using RNAiso Plus (9109, Takara, Shiga, Japan). Real-time PCR was performed using a Real-Time system (CFX96, Bio-Rad Laboratories, Inc., Hercules, CA, USA). The expression data were normalized to the geometric mean of the expression of the housekeeping gene GAPDH and calculated using the 2 − ΔΔCt method [30]. The primer sequences are listed in Additional file 1: Table S6.

### Patients, tissue specimens and immunohistochemistry (IHC) staining

This study was conducted on a total of 148 paraffin-embedded colon cancer specimens, which were acquired from patients diagnosed from 2006 to 2010. Patient consent and approval from the Institutional Research Ethics Committee (No. 2018003) were obtained to use these clinical materials for research purposes. The tumors were staged according to the 7th edition of the Cancer Stage Manual written by the American Joint Committee on Cancer (AJCC) [31]. A section of the excised tumor was analyzed by IHC and H&E staining as described previously [32].

### Plasmids, retroviral infection, and transfection

The human *SRPK1* cDNA was amplified by PCR and cloned into the pSin-EF2 lentiviral vector to construct the plasmids SW480-pSin-Vector, HCT-116-pSin-Vector, SW480-SRPK1, and HCT-116-SRPK1. Short hairpin RNA (shRNA)-mediated interference was used to knockdown *SRPK1* in SW480 and HCT-116 cells that cells by transfection with lentiviral constructs expressing the *SRPK1* shRNAs or control shRNA. The constructs (pSin-EF2-puro-Vector, pSin-EF2-puro-SRPK1, pLKO.1-puro-Vector, pLKO.1-puro-SRPK1-sh1#, and pLKO.1-puro-SRPK1-sh2#) were transfected (10 µg of each plasmid) into 2 × 10^6^ 293 T cells to generate retroviruses. The indicated stable cell lines were generated via retroviral infection and were selected for 10 days using 0.5 g/mL puromycin (S7417, Selleck, Houston, TX, USA), as described previously [33]. This resulted in SW480-pLKO.1-Vector, HCT-116-pLKO.1-Vector, SW480-SRPK1-sh1#, SW480-SRPK1-sh2#, HCT-116-SRPK1-sh1# and HCT-116-SRPK1-sh2# stable cell lines. The primer sequences can be found in Additional file 1: Table S5.

### Western blotting

Western blotting was performed as described previously [33]. Briefly, 50 mg of protein was subjected to sodium dodecyl sulfate polyacrylamide gel electrophoresis (SDS-PAGE) and transferred to polyvinylidene fluoride (PVDF) membranes (Millipore, Bedford, MA, USA). The membranes were blocked with 5% nonfat milk and anti-SRPK1 (611,072, BD Biosciences, San Jose, CA, USA), anti-Bcl-xL (BCL2 Like 1 long isoform) (ab32370, Abcam, Cambridge, UK), anti-Bcl-xS (BCL2 Like 1 short isoform) (124,266, Genetex, Waltham, MA, USA), anti-pro-PARP1 ( (ab32138, Abcam, Cambridge, UK), anti-cleaved PARP1 (poly(ADP-ribose) polymerase 1) (ab32064, Abcam), anti-p65 (8242, Cell Signaling Technology, Danvers, MA, USA), anti-IKKβ (A301-827A, Bethyl Laboratories, Montgomery, TX, USA), anti-IKKβ pY199 (ab59195, Abcam), anti-IκBα (ab32518, Abcam), anti-IκBα pS36 (ab133462, Abcam), anti-AKT (4694, Cell Signaling Technology), anti-AKT pS473 (4060, Cell Signaling Technology), anti- Histone H3 (4499, Cell Signaling Technology), anti-β-tubulin (ab210797, Abcam) and anti-GAPDH (glyceraldehyde-3-phosphate dehydrogenase) (A300-639A, Bethyl Laboratories) overnight at 4 °C. The membranes were then incubated with goat anti-rabbit (ab97051, Abcam) and anti-mouse (ab6789, Abcam) secondary antibodies for 1 h at room temperature, and then visualized and analyzed using the Amersham Imager 600 (Cytiva, Marlborough, MA, USA) software. The quantification of western blotting was performed by Image J (Version1.8.0, NIH, USA, https://imagej.nih.gov/ij/index.html) as pervious described [34]. Primary antibodies against β-tubulin and GAPDH were used as protein loading controls. The AKT inhibitor (S1078, Selleck) was used to verify the phosphorylation of IKK and IκB.

### Dual-luciferase reporter assay

Stable cell lines infected with retroviruses were seeded in DMEM supplemented with 10% FBS in triplicate in 24-well plates (5 × 10^4^ cells per well) and cultured for 12 h. The NF-kB luciferase reporter gene (pGL4.32 [luc2 NF-kB-RE Hyrgo], Promega, Madison, WI, USA) and pRL-TK Renilla plasmid were co-transfected into cells using Lipofectamine 3000 reagent (Invitrogen). At 48 h after transfection, dual luciferase reporter gene detection was performed using a Dual Luciferase Reporter Assay Kit (Promega) according to the manufacturer’s protocol [33].

### 3-(4,5-dimethyl-2-thiazolyl)-2,5-diphenyl-2H-tetrazolium bromide (MTT) assay

Cells were seeded in DMEM supplemented with 10% FBS in triplicate in 96-well plates (5 × 10^3^ cells per well) and cultured for 12 h. The medium was replaced with medium containing different concentrations of oxaliplatin and incubated for 24 h. According to CellTiter96® AQueous One Solution Cell Proliferation Assay Kit (Promega) instruction, the reagents were added to the culture medium, incubated for 1–4 h, and the absorbance value at 490 nm was read using a microplate reader. IC_50_ values were calculated using GraphPad Prism software (version 7, GraphPad Software, Inc., San Diego, CA, USA) via nonlinear regression (curve fit) using the oxaliplatin concentration *vs*. the normalized response (variable slope) method.

### Immunofluorescence assay

The immunofluorescence assay was performed as described previously [32]. Briefly, the cells were incubated with a primary monoclonal anti-p65 (1:100) antibody overnight at 4 °C and then incubated with a fluorescein-conjugated goat anti-rabbit secondary antibody (ab150077, Abcam) for 1 h at room temperature. The cells were visualized under a Dragonfly laser scanning confocal microscopy system (Andor, Belfast, UK).

### Terminal deoxynulceotidyl transferase nick-end-labeling (TUNEL) assay

Cells (5 × 10^4^) were seeded on coverslips and cultured for 24 h. Briefly, the cells were treated with the indicated concentrations of oxaliplatin for 24 h, fixed for 25 min in 4% paraformaldehyde, and washed twice with phosphate-buffered saline (PBS). The cells were then incubated with blocking solution for 10 min at room temperature. Following further washes with PBS, the cells were incubated on ice with 0.1% Triton X-100 (T8200, Solarbio, Beijing, China) in 0.1% sodium citrate (Tianjin Zhiyuan Chemical Reagent Co., Ltd., Tianjin, China) for 2 min. Subsequently, the cells were washed with PBS and incubated with 50 µL TUNEL reaction mixture containing the rTdT enzyme for 1 h at 37 °C. The TUNEL assay was performed according to the manufacturer’s instructions (G3250, Promega). After washing with PBS three times, the cells were incubated with 200 µL 4′,6-diamidino-2-phenylindole (DAPI) for 15 min in the dark, washed, and analyzed under an inverted light microscope (CKX53, Olympus, Tokyo, Japan).

### Nuclear extract preparation

The indicated cells were washed with 5 mL PBS containing a protease inhibitor cocktail (B14011, Bimake, Houston, TX, USA) and a phosphatase inhibitor cocktail (B15001, Bimake) before adding 3 mL ice-cold PBS containing the same protease and phosphatase inhibitor cocktails. The cells were transferred to a pre-chilled 15-mL conical tube and centrifuged for 5 min at 200×*g* at 4 °C. A Nuclear Extract kit (40,010, Active Motif, Rixensart, Belgium) was then used to isolate the nuclear extracts from the cell pellets, according to the manufacturer’s instructions.

### Statistical analysis

All statistical analyses were carried out using SPSS version 22.0 statistical software (IBM Corp., Armonk, NY, USA). Comparisons between groups were performed using the two-tailed Mann–Whitney U-test. The relationship between SRPK1 expression and clinicopathological characteristics was assessed using Spearman’s correlation test. Survival curves were plotted using the Kaplan–Meier method and compared using the log-rank test. Survival data were evaluated using uni- and multivariate Cox regression analyses. Bivariate correlations between variables were calculated using Spearman’s rank correlation coefficients. A p-value of less than 0.05 was considered statistically significant in all cases.

## Results

### SRPK1 is overexpressed and associated with clinical stage, TNM classification, and prognosis of survival in patients with colon cancer

**Fig. 1:**
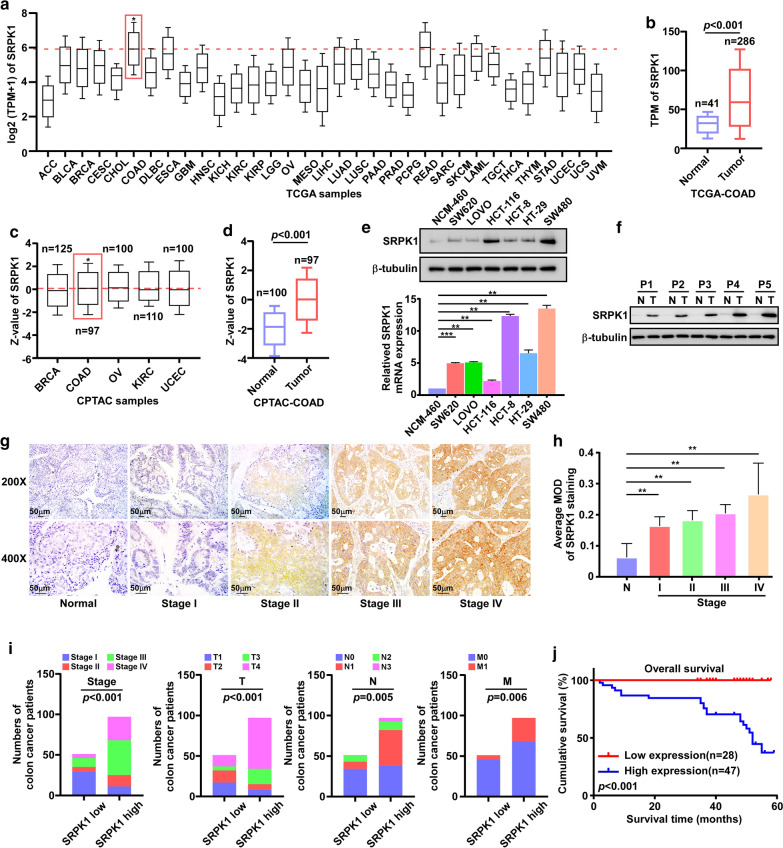
SRPK1 is overexpressed and associated with clinical stage, TNM classification, and prognosis of survival in patients with colon cancer. **a** SRPK1 mRNA expression analysis in TCGA pan-cancer samples. **b** SRPK1 mRNA expression analysis in TCGA colon cancer samples and normal tissues. **c** SRPK1 protein level analysis in CPTAC pan-cancer samples. **d** SRPK1 protein level analysis in CPTAC colon cancer samples and normal tissues. **e** The level of SRPK1 in colon cancer cell lines. **f** The level of SRPK1 in colon cancer patients’ tissues and normal tissues. **g** IHC analysis of SRPK1 in the tissues of patients with colon cancer. **h** A statistical analysis of the average MOD of SRPK1 staining (n = 10). **i** SRPK1 high (n = 97)/low (n = 51) expression is significantly associated with clinical stage and TNM classification of paraffin embedded sections from patients with colon cancer. **j** The correlation of SRPK1 levels and overall survival. **p < 0.01. *SRPK1* Serine-arginine protein kinase 1, *TCGA* The Cancer Genome Atlas, *CPTAC* Clinical Proteomic Tumor Analysis Consortium, *IHC * immunohistochemistry, *MOD* mean of optical density, *TNM* tumor-mode-metastasis

By analyzing the expression of SRPK1 in the Cancer Genome Atlas (TCGA) database, we found that the SRPK1 mRNA expression was the second highest in colon cancer, which was only lower than that in rectum adenocarcinoma (READ) among 33 types of cancer, and the SRPK1 mRNA expression was increased in the TCGA colon cancer tissues (n = 286) compared with that in normal tissues (n = 41) (p < 0.001) (Fig. 1a, b). In addition, the protein levels of SRPK1 in colon cancer were the second highest in the Clinical Proteomic Tumor Analysis Consortium (CPTAC) database, which was only lower than that in Ovarian serous cystadenocarcinoma (OV),and compared with that in normal tissues (n = 100), SRPK1 was also upregulated in CPTAC colon cancer tissues (n = 97) (p < 0.001) (Fig. 1c, d). Taken together, in colon cancer, SRPK1 was increased at both the mRNA and protein levels, and SRPK1 was highly expressed over other different cancer types. These findings suggested that SRPK1 might be a key oncoprotein in colon cancer.

Next, we confirmed the findings from the public databases in six colon cancer cell lines (Fig. 1e, Additional file 2: Fig. S1a ), five colon cancer tissues (Fig. 1f, Additional file 2: Fig. S1b) and 148 paraffin-embedded, archived colon cancer tissues (Fig. 1g). Statistical analyses of IHC-stained sections showed that SRPK1 was overexpressed with increasing clinical stage of colon cancer (Fig. 1h). These samples included 40 cases of stage I (27%), 20 cases of stage II (13.5%), 54 cases of stage III (36.5%), 34 cases of stage IV (23%), 25 cases of T1 (16.9%), 22 cases of T2 (14.8%), 24 cases of T3 (16.2%), 77 cases of T4 (52.1%), 72 cases of N0 (48.6%), 53 cases of N1 (35.8%), 18 cases of N2 (12.2%), 5 cases of N3 (3.4%), 114 cases of M0 (77%), and 34 cases of M1 (23%) (Fig. 1i and Table 1). SRPK1 protein expression was strongly associated with the clinical stage (p < 0.001), as well as the T (p < 0.001), N (p = 0.005), and M (p = 0.006) classifications (Fig. 1i and Additional file 1: Table S1). Spearman correlation analysis and univariate and multivariate analysis showed that SRPK1 protein expression correlated significantly with clinicopathological factors, including clinical stage and TNM classification (Additional file 1: Table S2 and S3).

**Table 1:** Clinicopathological characteristics of patients and expression of SRPK1 in colon cancer samples

Clinicopathological	No. (%)
Sex	
Male	83 (56.1)
Female	65 (43.9)
Age (years)	
≤ 60	47 (31.8)
> 60	101 (68.2)
Stage (AJCC)	
I	40 (27.0)
II	20 (13.5)
III	54 (36.5)
IV	34 (23.0)
T classification	
T_1_	25 (16.9)
T_2_	22 (14.9)
T_3_	24 (16.2)
T_4_	77 (52.0)
N classification	
N_0_	72 (48.6)
N_1_	53 (35.8)
N_2_	18 (12.2)
N_3_	5 (3.4)
M classification	
M_0_	114 (77.0)
M_1_	34 (23.0)
Survival (n = 75)	
Alive	51 (68.0)
Dead	24 (32.0)
Survival time of patients with low SRPK1 expression	
Mean	45.03
Median	47.00
Survival time of patients with high SRPK1 expression	
Mean	38.82
Median	46.00
Expression of SRPK1	
Low expression	51(34.5)
High expression	97(65.5)

**Fig. 2:**
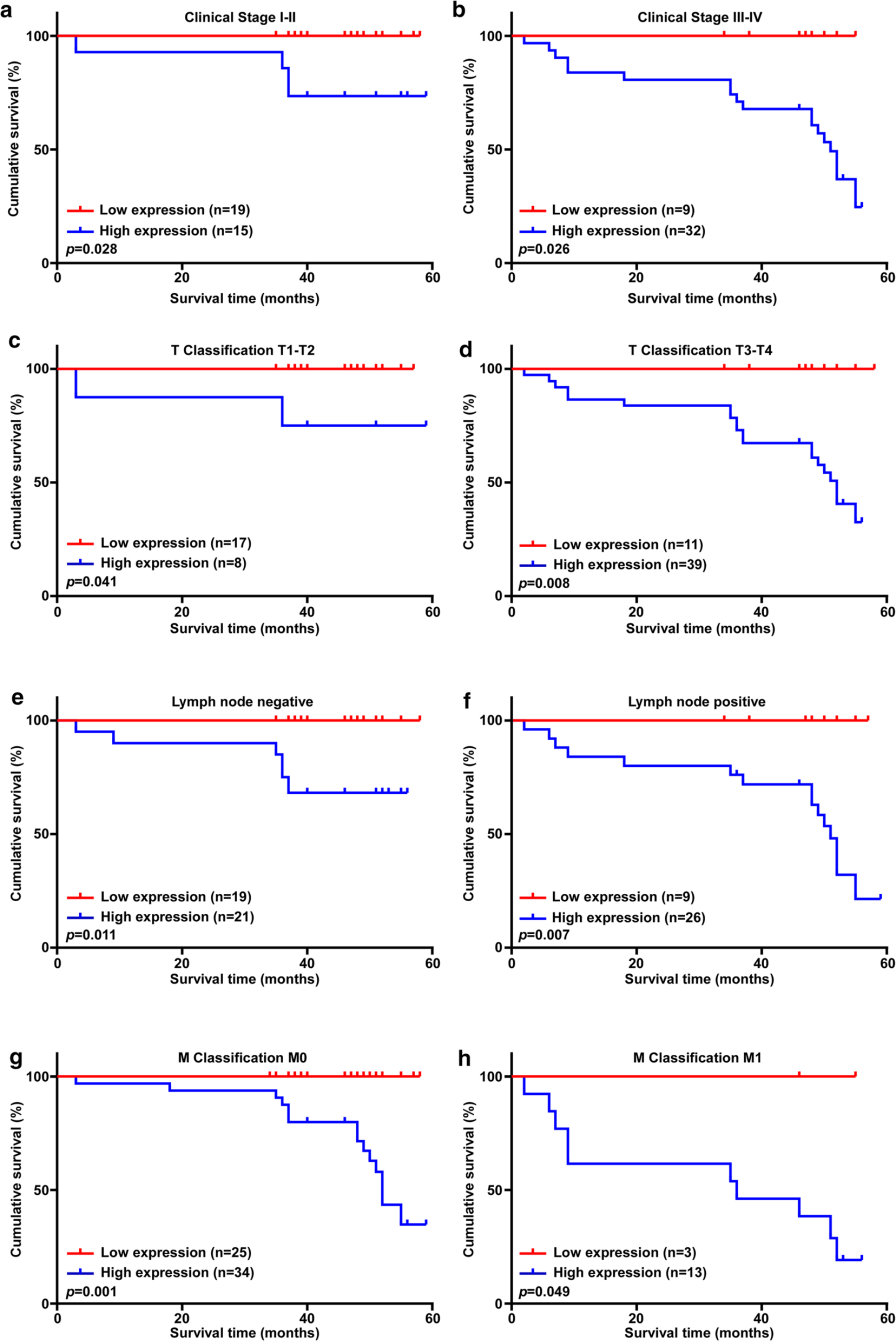
SRPK1 is associated with the prognosis of colon cancer clinical stage and TNM classification. **a** SRPK1 is associated with the prognosis of colon cancer clinical stage I–II. **b** SRPK1 is associated with the prognosis of colon cancer clinical stage III–IV. **c** SRPK1 is associated with the prognosis of colon cancer T1–T2 classification. **d** SRPK1 is associated with the prognosis of colon cancer T3–T4 classification. **e** SRPK1 is associated with the prognosis of lymph node negative colon cancer. **f** SRPK1 is associated with the prognosis of lymph node positive colon cancer. **g** SRPK1 is associated with the prognosis of M0 classification colon cancer. **h** SRPK1 is associated with the prognosis of M1 classification colon cancer.* SRPK1* Serine-arginine protein kinase 1, *TNM* tumor-mode-metastasis

Moreover, Kaplan–Meier analyses and log-rank tests demonstrated that the overall survival time of patients with high SRPK1 levels was significantly shorter than that of patients with low SRPK1 levels (p < 0.001; Fig. 1j). SRPK1 protein expression was associated negatively with survival time of stage I-II (p = 0.028; Fig. 2a), stage III–IV (p = 0.026; Fig. 2b), T1–T2 classification (p = 0.041; Fig. 2c), T3–T4 classification (p = 0.008; Fig. 2d), lymph node negative status (p = 0.011; Fig. 2e), lymph node positive status (p = 0.007; Fig. 2f), M0 classification (p = 0.001; Fig. 2g), and M1 classification (p = 0.049; Fig. 2h). In conclusion, our findings suggested that SRPK1 might be a prognostic factor for survival, which is closely connect to treatment of patients with colon cancer.

### Upregulated SRPK1 promotes the anti-apoptosis ability of colon cancer cell lines

**Fig. 3:**
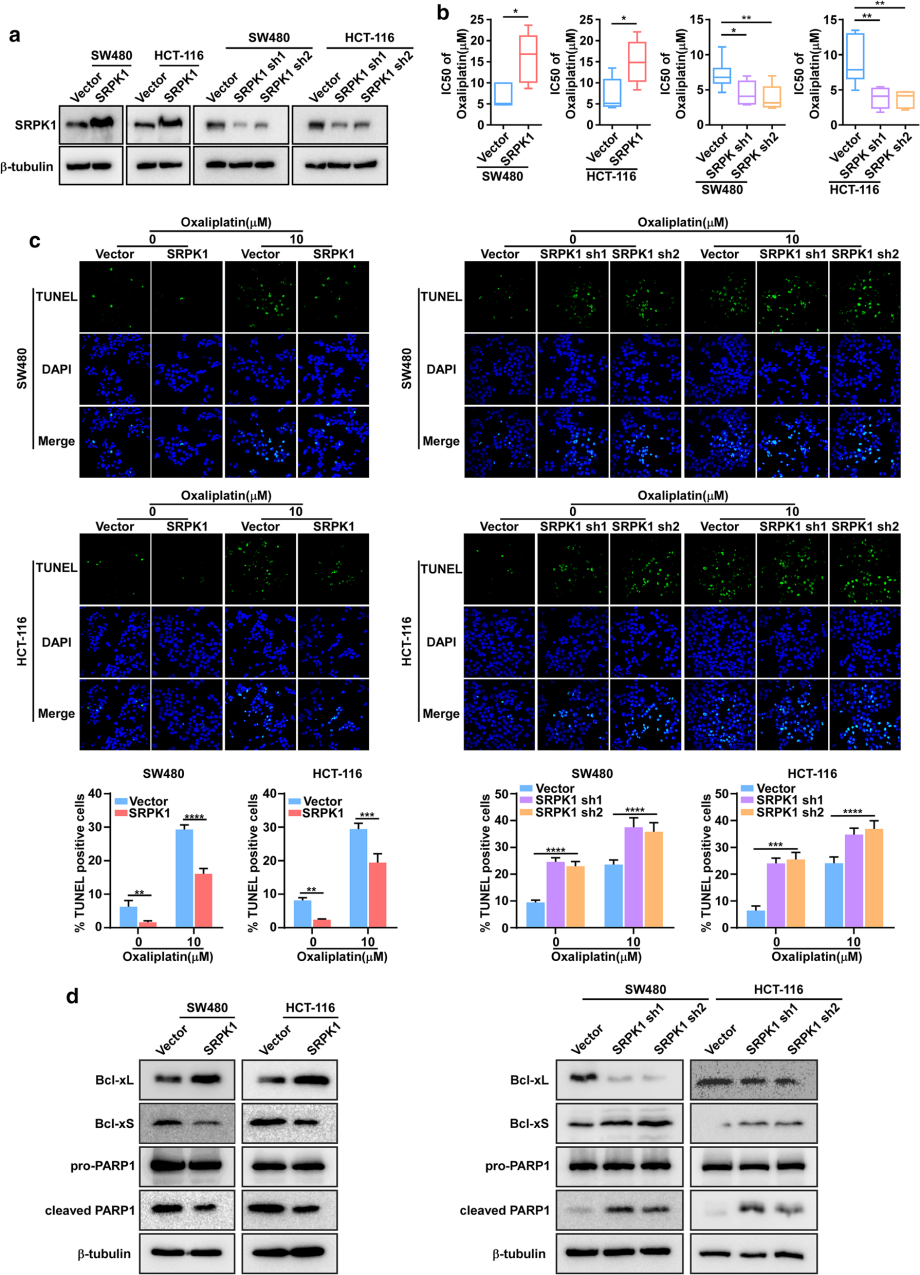
Upregulated SRPK1 promotes the ability of anti-apoptosis in colon cancer cell lines. **a** SRPK1 overexpression and downregulation in SW480 and HCT-116 cells were confirmed using western blotting. **b** MTT assay was examined and the IC_50_ was calculated in SW480 and HCT-116 cells overexpressing, or silenced for, SRPK1 and vector only with increasing oxaliplatin exposure (n = 6). **c** Representative TUNEL staining images of the indicated cells treated with increasing oxaliplatin concentrations, quantification of TUNEL^+^ cells in SW480 and HCT-116 cells overexpressing, or silenced for, SRPK1, and vector only with oxaliplatin treatment (n = 3). **d** Levels of Bcl-xL, Bcl-xS, and cleaved PARP1 in SW480 and HCT-116 cells overexpressing, or silenced for, SRPK1, and vector. β-tubulin was used as a loading control. *p < 0.05, **p < 0.01, ***p < 0.001. *SRPK1* Serine-arginine protein kinase 1, *TUNEL* terminal deoxynucleotidyl transferase nick-end-labeling, *Bcl-xL* BCL2 Like 1 long isoform, *Bcl-xS* BCL2 Like 1 short isoform, *PARP1* poly(ADP-ribose) polymerase 1

To determine the role of SRPK1 in colon cancer, we stably overexpressed or inhibited SRPK1 in SW480 and HCT-116 cells (Fig. 3a, Additional file 3: Fig. S2a). MTT assay was performed to evaluate the IC50 of oxaliplatin. SRPK1 overexpression increased the oxaliplatin IC_50_ value (Fig. 3b), whereas SRPK1 downregulation decreased the IC_50_ value (Fig. 3b). TUNEL assays also revealed that the percentage of TUNEL^+^ cell among SW480 and HCT-116 SRPK1 overexpressing cells decreased compared with that in vector-transduced cells at each concentration, whereas the opposite effect was obtained in SRPK1 knock-down SW480 and HCT-116 cells (Fig. 3c). These data indicated that SRPK1 overexpression in these cells exhibited an obvious anti-apoptosis effect (Fig. 3c). Consistently, SRPK1-knockdown led to apoptosis (Fig. 3c). Furthermore, we observed that and Bcl-xL levels increased, whereas Bcl-xS and cleaved PARP1 levels decreased, in SRPK1-overexpressing colon cancer cells (Fig. 3d, Additional file 3: Fig. S2b, c), and the opposite effect was observed in SRPK1 knockdown cell lines (Fig. 3d, Additional file 3: Fig. S2b, c). These results showed that elevated SRPK1 expression could enhance the anti-apoptosis capacity of colon cancer cells, whereas silencing SRPK1 expression enhanced apoptosis. Taken together, we demonstrated that SRPK1 increased anti-apoptosis capacity in colon cancer cell lines.

### SRPK1 increased the phosphorylation of AKT to activate the NF-κB pathway

**Fig. 4:**
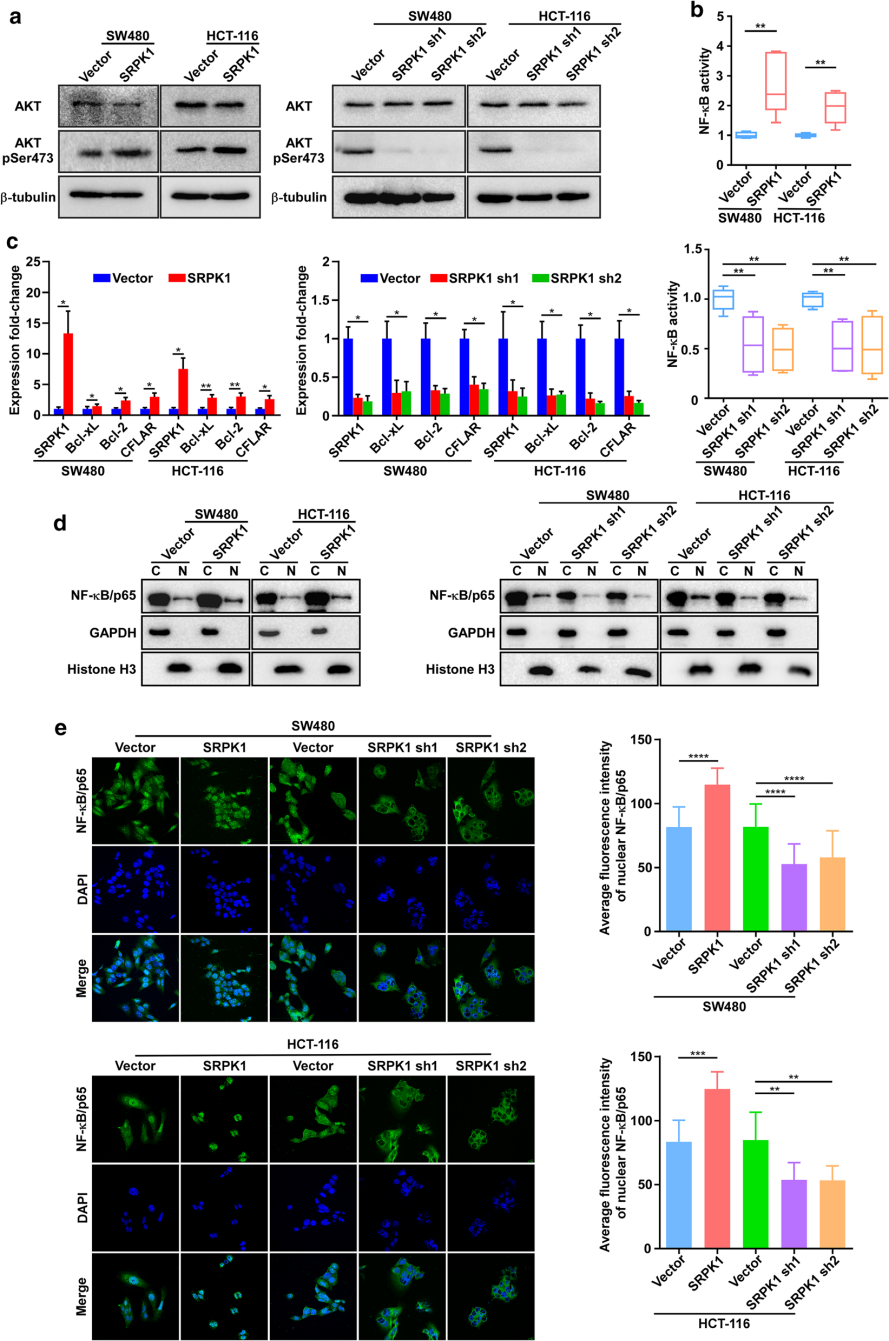
SRPK1 increased the phosphorylation of AKT to activate the NF-κB pathway. **a** Western blotting analysis the phosphorylation of AKT in SW480 and HCT-116 cells overexpressing SRPK1 or vector only, and downregulated for SRPK1, or vector only. **b** Luciferase assay to determine the transcriptional activity of NF-κB in SW480 and HCT-116 cells overexpressing, or silenced for, SRPK1, and vector only. **c** qPCR analysis of genes downstream of the NF-κB pathway in SW480 and HCT-116 cells overexpressing SRPK1 or vector only, and downregulated for SRPK1, or vector only. The bars indicate the means ± SD (n = 3). **d** Western blot analysis of the NF-κB p65 subunit in the nuclear and cytoplasmic fractions of SW480 and HCT-116 cells overexpressing SRPK1, or vector only, and downregulated for SRPK1, or vector only. **e**, Immunofluorescence analysis of p65 in SW480 and HCT-116 cells overexpressing SRPK1, or vector only, and downregulated for SRPK1, or vector only. *SRPK1* Serine-arginine protein kinase 1, * AKT* protein kinase B, *NF-κB* nuclear factor kappa B, *qPCR* quantitative real-time polymerase chain reaction

Both the in vitro and in vivo data presented above suggested that elevated levels of SRPK1 might enhance anti-apoptosis in colon cancer cells. Thus, we sought to determine the mechanisms underlying the anti-apoptosis effect. Fu et al. reported that SRPK1 could interact with AKT to promote its phosphorylation [35]. Thus, we speculated SRPK1 could be involved in colon cancer pathway activation by enhancing AKT phosphorylation. Indeed, the level of phosphorylated AKT was upregulated in SRPK1 overexpressing SW480 and HCT-116 cells, while SRPK1 silencing decreased the level of phosphorylated AKT (Fig. 4a, Additional file 4: Fig. S3a). The NF-κB pathway, which is directly downstream of AKT, had been reported to regulate proteins that inhibit apoptosis, which is associated markedly with cancer [24]. Here, we performed a luciferase reporter assay and found that SRPK1 overexpression increased the transcriptional activity of the NF-κB pathway in SW480 and HCT-116 cells, whereas, the opposite results were obtained in SRPK1-silenced SW480 and HCT-116 cells (Fig. 4b). Analysis by qPCR also revealed that the levels of NF-κB pathway downstream target genes were markedly increased in SW480 and HCT-116 SRPK1-overexpressing cells and reduced in SW480 and HCT-116 SRPK1-silenced cells (Fig. 4c), suggesting that SRPK1 increases AKT phosphorylation to promote NF-κB pathway activation.

Mechanistically, we further observed that SRPK1 overexpression promoted p65 nuclear translocation in SW480 and HCT-116 cells, whereas SRPK1 knockdown inhibited p65 nuclear translocation in SW480 and HCT-116 cells, compared with that in control cells (Fig. 4d, e, Additional file 4: Fig. S3b). In addition, we observed that oxaliplatin could decrease the level of phosphorylated AKT and the nuclear location of p65 (Additional file 5: Fig. S4). However, when the SRPK1 was overexpressed, oxaliplatin failed to regulate the level of phosphorylated AKT and p65 (Additional file 2: Fig. S1). These results indicated that SRPK1 could promote p65 translocation to activate the NF-κB pathway against oxaliplatin treatment.

### SRPK1 induces IκB phosphorylation dependent on AKT activation

**Fig. 5:**
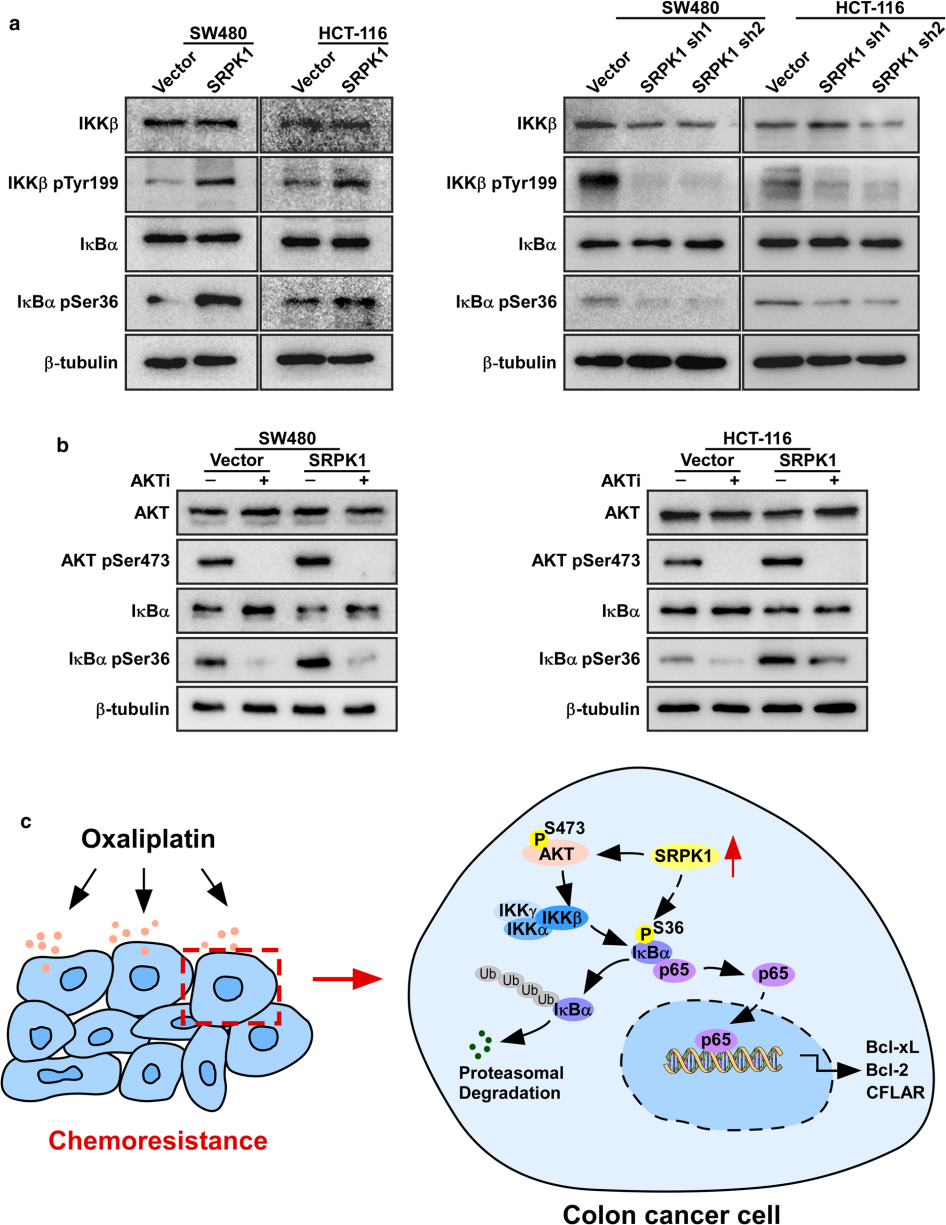
SRPK1 induced IκB phosphorylation dependent on AKT activation. **a** Western blotting analysis of the phosphorylation of IKKβ and IκBα of SW480 and HCT-116 cells overexpressing SRPK1 or vector only, and downregulated for SRPK1, or vector only. **b** Western blotting analysis the phosphorylation of AKT and IκBα of SW480 and HCT-116 cells overexpressing SRPK1, or vector only, under AKT inhibitor treatment. **c** A model of how SRPK1 enhances the anti-apoptosis ability of colon cancer via the NF-κB pathway by activating AKT. *p < 0.05, **p < 0.01, ***p < 0.001. *SRPK1* Serine-arginine protein kinase 1, *AKT* protein kinase B, *IκB* inhibitor of nuclear factor kappa B, *IKKβ* IκB kinase beta subunit, *IκBα* IκB alpha subunit, *NF-κB* nuclear factor kappa B

Among on NF-κB pathway, IKK is a downstream target of AKT, and when IKK and IκB are phosphorylated, NF-κB would be released, which activates the NF-κB pathway [26–28]. Thus, we detected the phosphorylation of IKKβ and IκBα. Immunoblotting analyses showed increased levels of phosphorylated IKKβ and IκBα in SRPK1 overexpressing SW480 and HCT-116 cells, whereas SRPK1 silencing decreased the levels of phosphorylated IKKβ and IκBα (Fig. 5a, Additional file 6: Fig. S5a, b). We then assessed whether SRPK1-mediated activation of the NF-κB pathway was AKT dependent. We demonstrated decreased levels of phosphorylated IκBα under AKT inhibitor treatment (Fig. 5b, Additional file 6: Fig. S5c, d) in cells overexpressing SRPK1 or control group, suggesting that SRPK1 activates the NF-κB pathway dependent on AKT. Collectively, these results indicated that elevated SRPK1 expression conferred an anti-apoptosis ability by activating the NF-κB pathway by increasing the phosphorylation of AKT in colon cancer (Fig. 5c).

## Discussion

In the present study, the expression of SRPK1 in paraffin-embedded tissues from 148 patients with colon cancer was greatly associated with clinical pathology stage and patient survival prognosis, which suggested that SRPK1 could be a potential biomarker and therapeutic target for colon cancer diagnosis, prognosis, and therapy.

SRPK1 is maintained in the cytoplasm with chaperone HSP70/HSP90 and translocates to the nucleus to perform alternative splicing [36]. Bcl-X is a well-known apoptosis regulator modulated by alternative splicing. Anti-apoptosis protein Bcl-xL and pro-apoptosis protein Bcl-xS are the two inverse function isoforms formed from the BCL2L1 (Bcl-X) gene alternative splicing process [37]. However, whether SRPK1 could work as an oncoprotein to induce apoptosis via activation of key pathways is unclear. In the present study, Bcl-xL translation levels increased consequent to SRPK1 expression levels, which suggest that SRPK1 might be involve in the apoptosis process by participating in upstream pathway activation, besides its role as an SR splicing factor of Bcl-X splicing.

Oxaliplatin represent a major class of chemotherapeutic drugs in cancer cells [2]. It has been reported that NF-κB pathway may play an important role in tumorigenesis and the level of activation of NF-κB pathway is associated with chemotherapy effect [38], suggesting that combined NF-κB inhibitor with chemotherapy drugs might further suppress colon cancer proliferation. In addition, SRPK1 promotes cancer by regulating AKT phosphatase to induce AKT dephosphorylation by interacting with PHLPP1, which could dephosphorylate p-AKT, lead to AKT constitutive activation suggesting that SRPK1 plays a key role in signaling transduction [35]. Taken together, we assumed that SRPK1 overexpression might increase AKT phosphorylation to induce NF-κB pathway activation to enhance the ability of anti-apoptosis in colon cancer cells against oxaliplatin treatment.

Our data showed that after oxaliplatin treatment, the phosphorylation of AKT and the accumulation of NF-κB were both decreased in SW480 cell, this phenomenon was not observed in SRPK1 overexpression stable cell lines, suggesting that SRPK1 could maintain the AKT phosphorylation and the accumulation of NF-κB under oxaliplatin treatment (Additional file 2: Fig. S1). Furthermore, the data presented here show that SRPK1 participates in the activation of the NF-κB pathway by increasing IKK phosphorylation. IKK is a downstream target of AKT, which consists of the kinase subunits IKKα and IKKβ. Only when IKK phosphorylates IκB, could NF-κB be release from IκB’s binding to accumulate in the nucleus and execute its biological function of increasing the transcription of its downstream target genes, including anti-apoptosis genes [26–28]. We found that the levels of phosphorylated AKT, IKK, and IκB increased in SRPK1 overexpressing colon cancer cell lines, which contribute to the activation of the downstream NF-κB pathway. These findings showed that SRPK1 could transduce anti-apoptosis signals directly as a downstream target of AKT to activate the NF-κB pathway, which suggested that SRPK1 participates in the anti-apoptosis process via the NF-κB pathway by activating AKT. Therefore, identifying new molecules that target in NF-κB pathway might contribute to overcoming chemoresistance, which is an on-going project in our laboratory. Furthermore, targeting SRPK1’s kinase activity would not suppress tumorigenesis completely; therefore, SRPK1’s non-splicing-kinase activity should be considered in clinical treatment, which will help to better understanding chemoresistance.

## Conclusions

Our finding suggested that SRPK1 enhances the anti-apoptosis ability of colon cancer via the NF-κB pathway by activating AKT. In addition, specific inhibition of SRPK1 might represent a potential anti-drug resistance therapy, and SRPK1 might also be a prognostic biomarker for oxaliplatin resistance in colon cancer. Targeting SRPK1 might enhance the sensitivity of patients to oxaliplatin.

## Supplementary Information

**Additional file 1: Table S1.** Correlation between the clinicopathological features and SRPK1 expression. **Table S2.** Spearman correlation analysis between SRPK1 expression and clinicopathological factors. **Table S3.** Univariate and multivariate analysis of different prognostic parameters in patients with colon cancer. **Table S4.** Univariate and multivariate analysis of different prognostic parameters in patients with colon cancer. **Table S5.** Primers used for plasmid construction. **Table S6.** Primers used for real-time PCR.

**Additional file 2: Fig. S1** The quantification of SRPK1 expression. **a** The quantification of SRPK1 expression of Fig. 1e. **b** The quantification of SRPK1 expression of Fig. 1f.

**Additional file 3: Fig. S2** The quantification of western blotting from Fig. 3. **a** The quantification of SRPK1 expression of Fig. 3a. **b** The quantification of Bcl-xS/xL expression of Fig. 3d. **c** The quantification of cleaved/pro-PARP1 expression of Fig. 3d.

**Additional file 4: Fig. S3** The quantification of western blotting from Fig. 4. **a** The quantification of pAKT/AKT expression of Fig. 4a. **b** The quantification of p65 expression in nuclear of Fig. 4d.

**Additional file 5: Fig. S4** SRPK1 promoted p65 nuclear translocation under oxaliplatin treatment. **a** Western blotting analysis the phosphorylation of AKT in SW480 under oxaliplatin treatment. **b** Western blot analysis of the NF-κB p65 subunit in the nuclear and cytoplasmic fractions of SW480 after oxaliplatin treatment. **c** The quantification of pAKT/AKT expression of a. **d** The quantification of p65 expression in nuclear of b.

**Additional file 6: Fig. S5** The quantification of western blotting from Fig. 5. **a** The quantification of pIKKβ/IKKβ expression of Fig. 5a. **b** The quantification of pIκBα/IκBα expression of Fig. 5a. **c** The quantification of pAKT/AKT expression of Fig. 5b under AKT inhibitor treatment. **d** The quantification of pIκBα/IκBα expression of Fig. 5b under AKT inhibitor treatment.

## Data Availability

The datasets used and/or analysed during the current study are available from the corresponding author on reasonable request.

## Abbreviations

AJCC: American Joint Committee on Cancer
AKT: AKT serine/threonine kinase 1
ATCC: American Type Culture Collection
Bcl-2: B cell leukemia/lymphoma 2
Bcl-xL: BCL2 like 1 long isoform
Bcl-xS: BCL2 like 1 short isoform
cDNA: Complimentary Deoxyribonucleic acid
CFLAR: CASP8 and FADD like apoptosis regulator
CPTAC: Clinical Proteomic Tumor Analysis Consortium
Ct: Cycle threshold
DAPI: 4',6-Diamidino-2-phenylindole
DMEM: Dulbecco’s Modified Eagle Medium
DMSO: Dimethyl sulfoxide
EGFR: Epidermal Growth Factor Receptor
FBS: Fetal Bovine Serum
GAPDH: Glyceraldehyde-3-phosphate dehydrogenase
H3: Histone H3
IC50: Half inhibitory concentration
IHC: Immunohistochemistry
IKKβ: Inhibitor of nuclear factor kappa B kinase subunit beta
IKKα: Component of inhibitor of nuclear factor kappa B kinase complex
IκBα: NF-kB inhibitor alpha
μg: Microgram
mg: Milligrams
mL: Milliliter
mRNA: Messenger Ribonucleic Acid
MTT: 3-(4,5-Dimethyl-2-thiazolyl)-2,5-diphenyl-2H-tetrazolium bromide
NF-κB: Nuclear factor kappa B
OV: Ovarian serous cystadenocarcinoma
p65: RELA proto-oncogene, NF-kB subunit
PARP1: Poly(ADP-ribose) polymerase 1
PBS: Phosphate Buffer Saline;
PCR: Polymerase chain reaction
PHLPP: Pleckstrin homology (PH) domain leucine-rich repeat protein phosphatase
PI: Propidium Iodide
Puro: Puromycin
RNA: Ribonucleic acid
shRNA: Short hairpin Ribonucleic acid
SRSF: Serine-arginine (SR) rich splicing factors
TCGA: Cancer Genome Atlas
