# Supplementary material for: SRPK1/AKT axis promotes oxaliplatin-induced anti-apoptosis via NF-κB activation in colon cancer
Source: J Transl Med. 2021 Jun 30;19:280. doi: 10.1186/s12967-021-02954-8 (PMC8243872; doi:10.1186/s12967-021-02954-8)
Supplement: Supplementary file 1 — Additional file 1: Table S1. Correlation between the clinicopathological features and SRPK1 expression. Table S2. Spearman correlation analysis between SRPK1 expression and clinicopathological factors. Table S3. Univariate and multivariate analysis of different prognostic parameters in patients with colon cancer. Table S4. Univariate and multivariate analysis of different prognostic parameters in patients with colon cancer. Table S5. Primers used for plasmid construction. Table S6. Primers used for real-time PCR. [file 12967_2021_2954_MOESM1_ESM.docx]

| **Supplementary table 1** Correlation between the clinicopathological features and SRPK1 expression | | | |
| --- | --- | --- | --- |
| Characteristics | SRPK1 | | *p* value |
|  | low | high |  |
| Sex |  |  | 0.060 |
| Male | 34 | 49 |  |
| Female | 17 | 48 |  |
| Age (years) |  |  | 0.158 |
| ≤ 60 | 20 | 27 |  |
| > 60 | 31 | 70 |  |
| Stage (AJCC) |  |  | < 0.001 |
| I | 29 | 11 |  |
| II | 6 | 14 |  |
| III | 11 | 43 |  |
| IV | 5 | 29 |  |
| T classification |  |  | < 0.001 |
| T_1_ | 17 | 8 |  |
| T_2_ | 15 | 7 |  |
| T_3_ | 5 | 19 |  |
| T_4_ | 14 | 63 |  |
| N classification |  |  | 0.005 |
| N_0_ | 34 | 38 |  |
| N_1_ | 9 | 44 |  |
| N_2_ | 7 | 11 |  |
| N_3_ | 1 | 4 |  |
| M classification |  |  | 0.006 |
| M_0_ | 46 | 68 |  |
| M_1_ | 5 | 29 |  |
| Survival (n = 75) |  |  | < 0.001 |
| Alive | 27 | 24 |  |
| Dead | 1 | 23 |  |

| **Supplementary table 2** Spearman correlation analysis between SRPK1 expression and clinicopathological factors | | |
| --- | --- | --- |
| Variables | SRPK1 | |
|  | Spearman Correlation | *p* Value |
| Sex | 0.155 | 0.061 |
| Age | 0.116 | 0.160 |
| Clinical stage | 0.445 | < 0.001 |
| T classification | 0.432 | < 0.001 |
| N classification | 0.221 | 0.010 |
| M classification | 0.227 | 0.006 |
| Survival | -0.221 | < 0.001 |

| **Supplementary table 3** Univariate and multivariate analysis of different prognostic parameters in patients with colon cancer | | | | | |  |
| --- | --- | --- | --- | --- | --- | --- |
| Variables | Univariate analysis | | Multivariate analysis | | |  |
|  | *p* | Regression coefficient (SE) | *p* | Reative risk | 95% confidence interval | |
| Clinical stage | 0.027 | 0.468 (0.221) | 0.014 | 2.450 | 0.949–2.204 | |
| Expression of SRPK1 | 0.016 | 1.085 (0.449) | 0.047 | 1.348 | 1.002–1.307 | |
| T classification | 0.047 | 0.426 (0.663) | 0.020 | 1.011 | 0.431–2.376 | |
| N classification | 0.003 | 0.500(0.283) | 0.019 | 1.022 | 0.474–2.200 | |
| M classification | 0.043 | 0.508(0.451) | 0.043 | 0.585 | 0.476–4.473 | |
| Age | 0.015 | 1.197 (0.740) | 0.048 | 4.401 | 0.952–20.353 | |

| **Supplementary table 4** STR profiles of cell lines | | | | | | |  |
| --- | --- | --- | --- | --- | --- | --- | --- |
| Cell lines  STR loci | SW480 | HCT-116 | LOVO | HT-29 | HCT-8 | SW620 | |
|  |  |  |  |  |  |  |  |
| D5S818 | 13,13 | 10,11 | 11,13 | 11,12 | 13,13 | 13,13 | |
| D13S317 | 12,12 | 10,12 | 8,11 | 11,11 | 8,11 | 12,12 | |
| D7S820 | 8,8 | 11,12 | 9.3,10,11 | 10,10 | 10,11.3 | 8,9 | |
| D16S539 | 13,13 | 11,13 | 9,12 | 11,12 | 12,13 | 9,13 | |
| VWA | 16,16 | 17,22 | 17,18 | 17,19 | 18,19 | 16,16 | |
| TH01 | 8,8 | 8,9 | 9.3,9.3 | 6,6 | 7,9.3 | 8,8 | |
| AM | X,X | x,y | X,Y | x,x | X,X | X,X | |
| TPOX | 11,11 | 8,8 | 8,9 | 8,9 | 8,11 | 11,11 | |
| CSF1PO | 13,14 | 7,10 | 10,11,13,14 | 11,12 | 12,12 | 13,14 | |
| Matched to | SW480 | HCT-116 | LOVO | HT-29 | HCT-8 | SW620 | |

**Supplementary table 5** Primers used for plasmid construction

| Gene | Primers (5′-3′) |
| --- | --- |
| *SRPK1* | Forward: ATGGAGCGGAAAGTGCTTGC |
|  | Reverse: TTAGGAGTTAAGCCAAGGGTGC |

**Supplementary table 6** Primers used for real-time PCR

| Gene | Primers (5′-3′) |
| --- | --- |
| *SRPK1* | Forward: ATGGAGCGGAAAGTGCTTG |
|  | Reverse: GAGCCTCGGTGCTGAGTTT |
| *BCL-xL* | Forward: GAGCTGGTGGTTGACTTTCTC |
|  | Reverse: TCCATCTCCGATTCAGTCCCT |
| *BCL2* | Forward: GGTGGGGTCATGTGTGTGG |
|  | Reverse: CGGTTCAGGTACTCAGTCATCC |
| *CFLAR* | Forward: TCAAGGAGCAGGGACAAGTTA |
|  | Reverse: GACAATGGGCATAGGGTGTTATC |
