# Supplementary figures and images for: SRPK1/AKT axis promotes oxaliplatin-induced anti-apoptosis via NF-κB activation in colon cancer
Source: J Transl Med. 2021 Jun 30;19:280. doi: 10.1186/s12967-021-02954-8 (PMC8243872; doi:10.1186/s12967-021-02954-8)

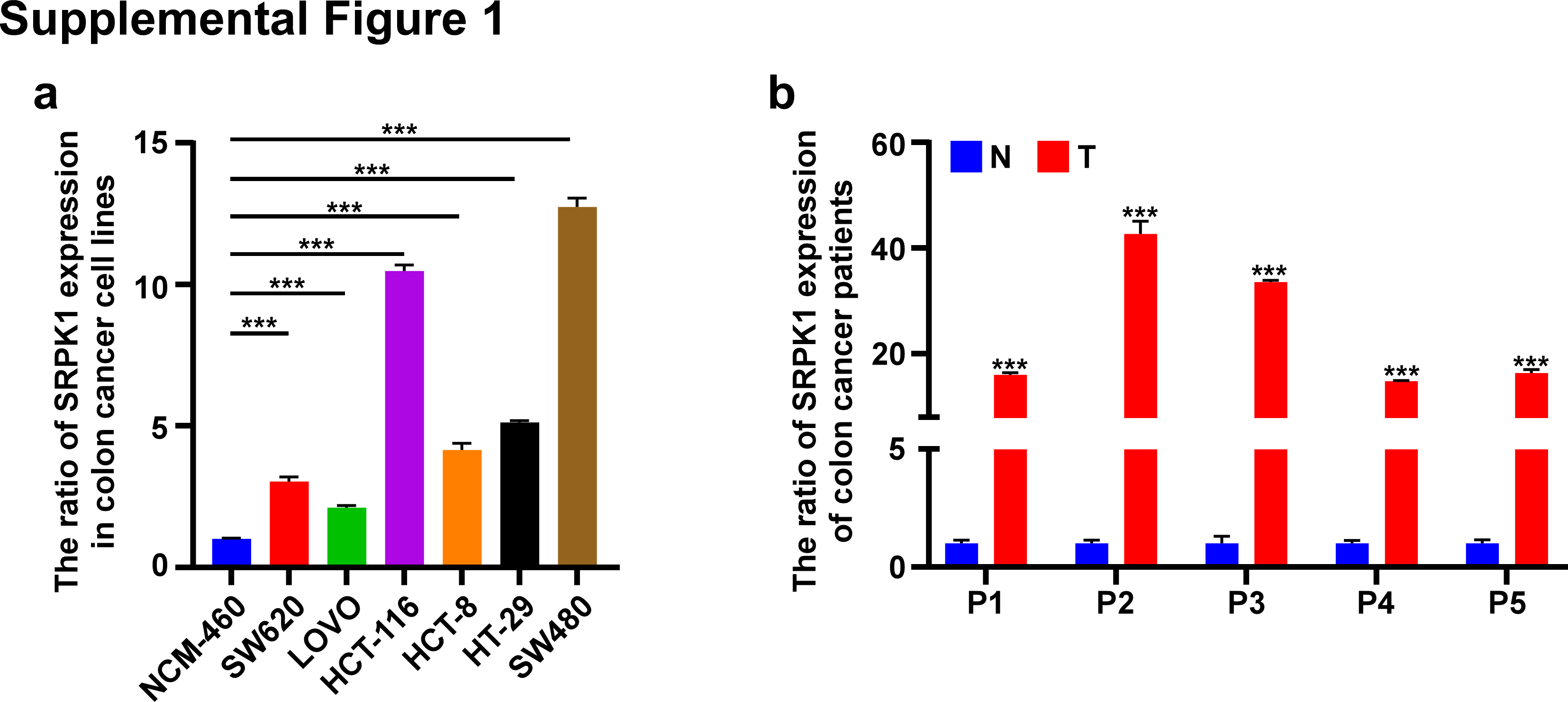

Supplement: Supplementary file 2 — Additional file 2: Fig. S1 The quantification of SRPK1 expression. a The quantification of SRPK1 expression of Fig. 1e. b The quantification of SRPK1 expression of Fig. 1f. [file 12967_2021_2954_MOESM2_ESM.tif]

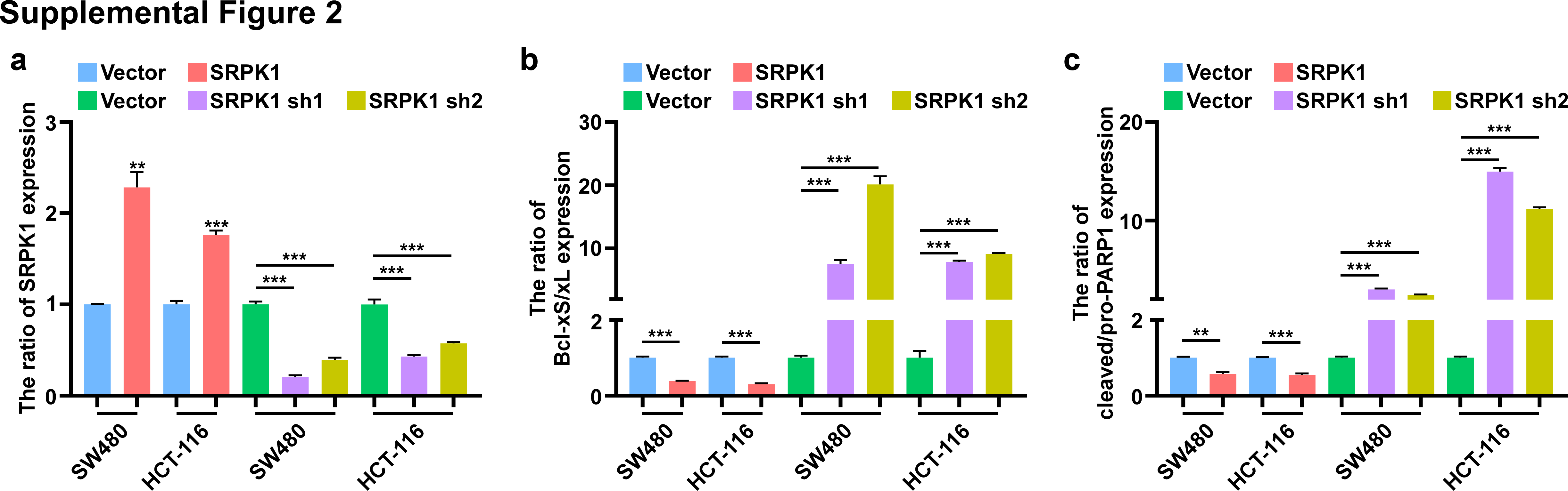

Supplement: Supplementary file 3 — Additional file 3: Fig. S2 The quantification of western blotting from Fig. 3. a The quantification of SRPK1 expression of Fig. 3a. b The quantification of Bcl-xS/xL expression of Fig. 3d. c The quantification of cleaved/pro-PARP1 expression of Fig. 3d. [file 12967_2021_2954_MOESM3_ESM.tif]

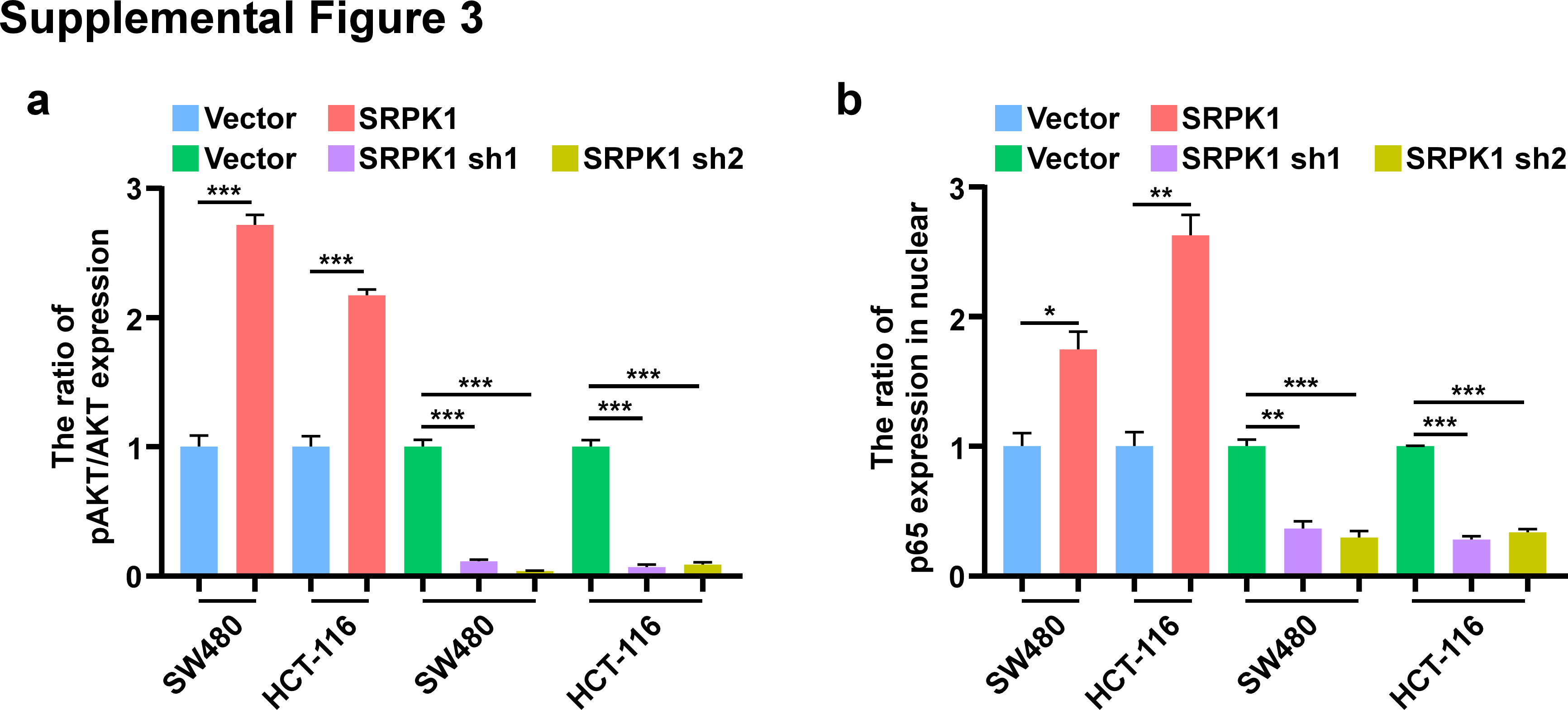

Supplement: Supplementary file 4 — Additional file 4: Fig. S3 The quantification of western blotting from Fig. 4. a The quantification of pAKT/AKT expression of Fig. 4a. b The quantification of p65 expression in nuclear of Fig. 4d. [file 12967_2021_2954_MOESM4_ESM.tif]

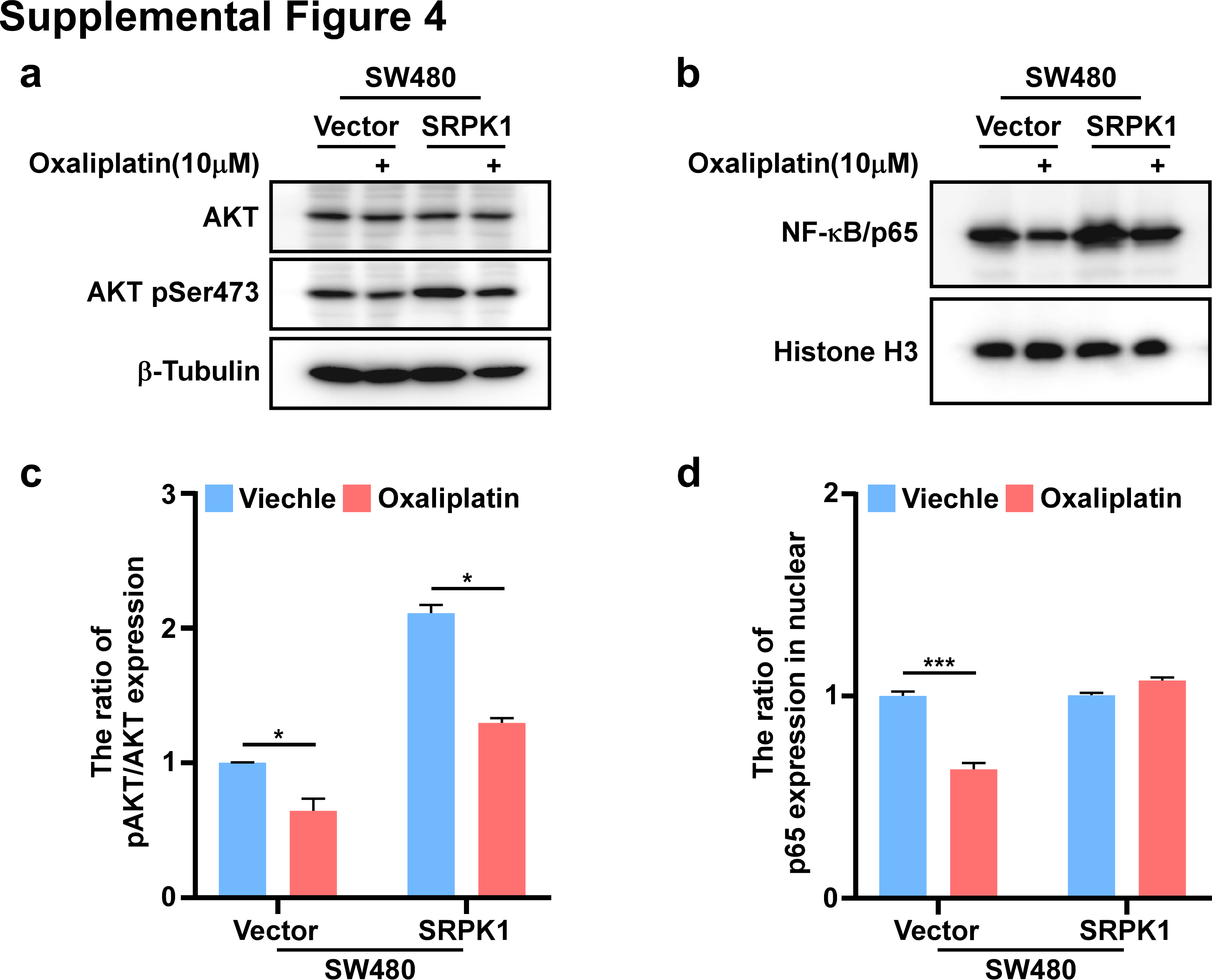

Supplement: Supplementary file 5 — Additional file 5: Fig. S4 SRPK1 promoted p65 nuclear translocation under oxaliplatin treatment. a Western blotting analysis the phosphorylation of AKT in SW480 under oxaliplatin treatment. b Western blot analysis of the NF-κB p65 subunit in the nuclear and cytoplasmic fractions of SW480 after oxaliplatin treatment. c The quantification of pAKT/AKT expression of a. d The quantification of p65 expression in nuclear of b. [file 12967_2021_2954_MOESM5_ESM.tif]

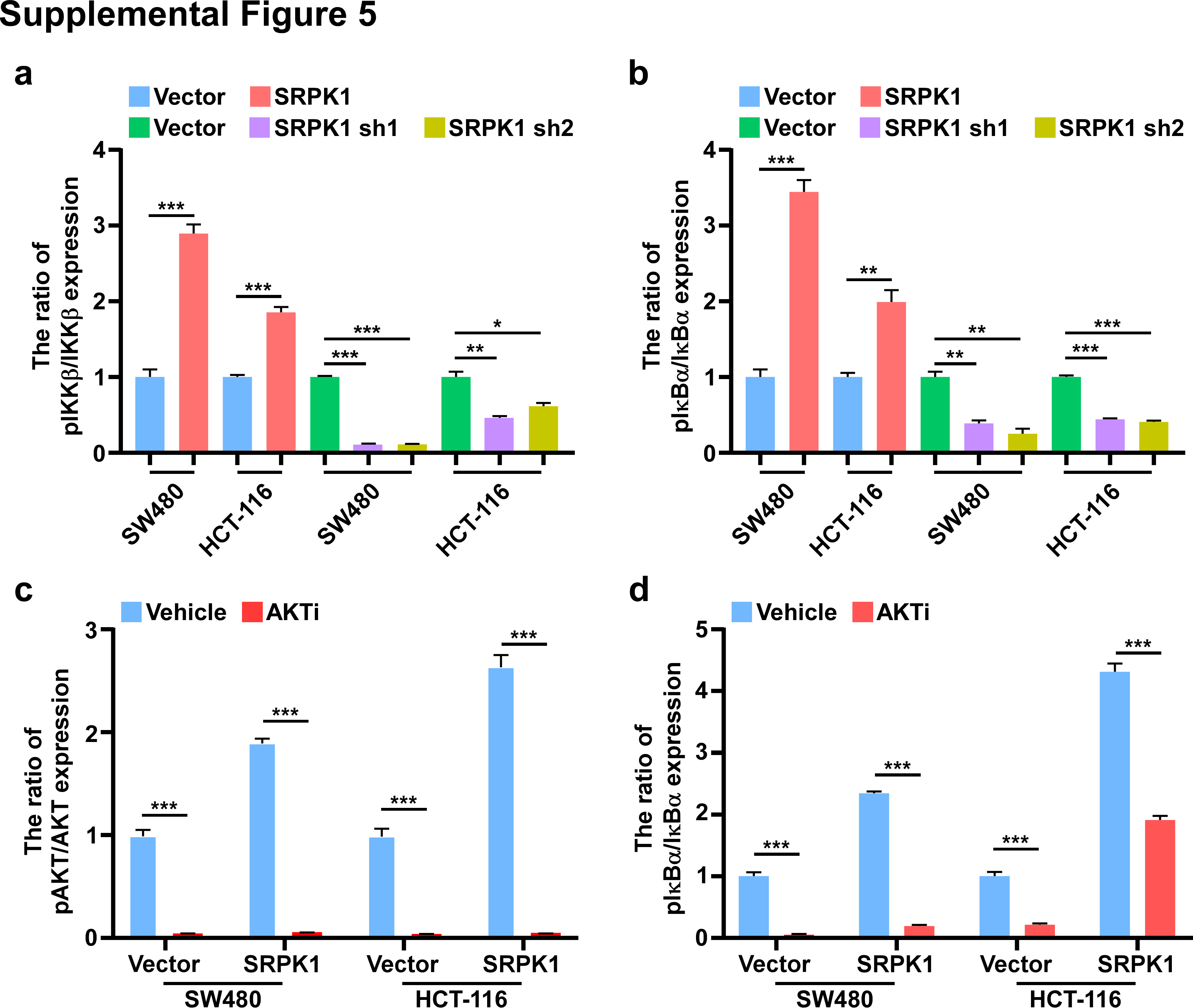

Supplement: Supplementary file 6 — Additional file 6: Fig. S5 The quantification of western blotting from Fig. 5. a The quantification of pIKKβ/IKKβ expression of Fig. 5a. b The quantification of pIκBα/IκBα expression of Fig. 5a. c The quantification of pAKT/AKT expression of Fig. 5b under AKT inhibitor treatment. d The quantification of pIκBα/IκBα expression of Fig. 5b under AKT inhibitor treatment. [file 12967_2021_2954_MOESM6_ESM.tif]
